# Asciminib and inotuzumab ozogamicin: a new combination for highly refractory Ph-positive B-cell acute lymphoblastic leukemia

**DOI:** 10.1016/j.lrr.2025.100546

**Published:** 2025-09-16

**Authors:** Aneta Strumilowska, Katherine Devitt, Joanna Conant, Juli-Anne Gardner, Ashley Volaric, Neel Hegde, Diego Adrianzen-Herrera

**Affiliations:** a Department of Medicine, Division of Hematology and Oncology, University of Vermont Larner College of Medicine, Burlington, VT, USA; b Department of Pathology and Laboratory Medicine, University of Vermont Larner College of Medicine, Burlington, VT, USA

To the editor,

Approximately 20–30 % of adults with B-cell acute lymphoblastic leukemia (B-ALL) exhibit the Philadelphia chromosome translocation (Ph) and express the BCR::ABL1 tyrosine kinase. The frequency of Ph-positive B-ALL is greater in older populations, accounting for up to 50 % in elderly patients [1]. Ph-positive B-ALL is a biologically distinct subtype associated with poorer prognosis, reduced response to chemotherapy, and higher relapse rate [2]. Tyrosine kinase inhibitors (TKIs) have improved outcomes and remission rates [2], however long term disease control remains difficult as relapse results from acquired ABL1 kinase domain (KD) mutations or KD-independent causes of TKI resistance. Over 80 % of adults with relapsed disease have KD mutations hindering TKI binding [3]. Challenging mutations as T315I confer resistance against most TKIs except the third generation ponatinib [4]. However ponatinib is not without limitations, having an adverse safety profile in older adults, who make up a large portion of Ph-positive B-ALL cases, and not addressing resistance from KD-independent mechanisms [5], occurring without detectable TKI mutations [3].

Asciminib is a first-in-class Specifically Targeting the ABL Myristoyl Pocket (STAMP) inhibitor of BCR::ABL1 that potently inhibits ABL1 kinase activity via allosteric binding to the myristoyl pocket in a non-ATP-competitive manner. It has demonstrated activity against both wild-type and mutant forms of BCR::ABL1, being effective against most KD mutations that confer resistance to ATP-competitive TKIs, including T315I. Asciminib is currently approved in Ph-positive chronic myeloid leukemia and represents a potential partner to therapeutic strategies in relapsed and refractory Ph-positive B-ALL patients. The bispecific T-cell engager (BiTE) blinatumomab which binds CD19 and CD3, the humanized CD22-directed monoclonal antibody-drug conjugate (ADC) inotuzumab ozogamicin, and chimeric antigen receptor (CAR) T-cells targeting CD19 are often used in combination with TKIs, particularly ponatinib, with demonstrated efficacy in relapsed and refractory Ph-positive B-ALL [6,7]. Asciminib may thus be a useful addition to these therapies in patients who have exhausted most or all ATP-pocket binding TKIs currently in use.

A 76-year-old man with past medical history of hypertension and coronary artery disease was diagnosed with B-ALL in 2018. Bone marrow biopsy revealed CD20-negative Ph-positive B-ALL with 92 % involvement. Cytogenetics and FISH confirmed t(9;22)(q34.1;q11.2) and BCR::ABL1 p190 transcript. Molecular data showed no IKZF1, CDKN2A/B or PAX5. He was treated with a modified Dana-Farber Cancer Institute (DFCI) 91–01 regimen [8] in combination with dasatinib, achieving measurable residual disease (MRD)-negative complete response (CR) following intensification phase, with absent detectable lymphoblasts by high sensitivity flow cytometric immunophenotyping (0.002 % sensitivity) and undetectable BCR::ABL1 p190 transcript (0.001 % sensitivity). He completed 8 cycles of the maintenance phase through 2019, but chemotherapy was held in July 2019 due to adverse events. Soon after, he developed pleural and pericardial effusions prompting discontinuation of dasatinib and transition to imatinib by January 2020.

By December 2020, he had rising BCR::ABL p190 transcript and bone marrow biopsy demonstrated Ph-positive ALL in first relapse, with CD19 bright expression by flow cytometry. Sequencing showed ABL1 A397P variant concerning for resistance to TKIs. Baseline clonotyping for next-generation sequencing (NGS) MRD monitoring was obtained. He was treated with ponatinib, starting at a dose of 30 mg and later increased to 45 mg daily, and steroids followed by blinatumomab, achieving MDR-negative CR following two cycles. Unfortunately, his course was complicated by myocardial infarction (MI) and peripheral artery disease prompting transition from ponatinib to bosutinib by August 2021. Close monitoring then revealed rising BCR::ABL p190 transcript by January 2022 and bone marrow biopsy confirmed second relapse, again with CD19 bright expression by flow cytometry. He underwent lymphodepleting chemotherapy with fludarabine and cyclophosphamide followed by CD19-directed CAR T-cell therapy with brexucabtagene autoleucel (brexu-cel) infusion on March 2, 2022, complicated by grade 1 cytokine release syndrome and grade 3 immune effector cell-associated neurotoxicity syndrome. Bosutinib was resumed after brexu-cel infusion and by July 2022, he achieved MRD-negative CR by clonality sequencing (0.00001 % sensitivity), undetectable BCR::ABL by qPCR, and absent phenotypic evidence of ALL by high sensitivity flow cytometric immunophenotyping.

The patient did well until October 2023, when BCR::ABL1 p190 transcript levels became detectable. Bone marrow biopsy by December 2023 demonstrated Ph-positive B-ALL in third relapse, involving 90 % of marrow cellularity with FISH 91.5 % of cells carrying the BCR::ABL1 translocation and positive CD22 expression on flow cytometry. KD mutation screen showed no BCR::ABL1 mutations associated with TKI-resistance. His relapse clinical course was complicated by rapidly progressing transfusion-dependent cytopenia, worsening chronic kidney disease, and non-ST elevation MI. At 82 years of age and with a complex medical history, he was found to be a poor candidate for cytotoxic chemotherapy or allogeneic hematopoietic stem cell transplantation (HSCT). Instead, therapy with a novel combination of inotuzumab ozogamicin and asciminib was initiated in January 2024, in 28-day treatment cycles. In cycle 1, he received inotuzumab ozogamicin at 0.8 mg/m2 on day 1 and 0.5 mg/m2 on days 8 and 15, in combination with asciminib 80 mg daily on days 1 through 28. A bone marrow biopsy on day 21 showed he achieved CR with MRD positivity at 0.033 % by multiparameter flow cytometry and 0.15 % detectable p190 transcript. For subsequent cycles, inotuzumab ozogamicin was dosed at 0.5 mg/m2 on day 1, 8 and 15, in combination with daily asciminib 80 mg. Following cycle 2, as of March 2024, he achieved MRD-negative CR, with absence of detectable disease demonstrated by B-cell clonality NGS sequencing, multiparameter flow cytometry and undetectable BCR::ABL p190 transcript by qPCR. His course was complicated by severe neutropenia and thrombocytopenia requiring delays of 7 and 14 days for cycles 3 and 4, and febrile neutropenia during cycle 4, but there was no evidence of hepatic venous occlusive disease. A bone marrow biopsy in July 2024 to study the cytopenia etiology revealed sustained MRD-negative remission. Inotuzumab ozogamicin was held after 4 cycles and the patient remained on asciminib monotherapy. He remains doing clinically well, and bone marrow biopsy MRD-negativity has been confirmed sequentially.

Limited data are available on the use of asciminib in Ph-positive B-ALL. A recently published phase 1 study reported promising outcomes of dual BCR::ABL1 inhibition in previously untreated patients receiving asciminib, dasatinib and prednisone [9]. However, its role in relapsed and refractory disease, especially after failure of conventional TKIs, remains to be determined. A handful of cases have reported effective use of asciminib [[10], [11], [12]], all of which combined it with ponatinib, motivated by the STAMP inhibitor being found to synergistically enhance target inhibition of ATP-pocket binding TKIs and thus constituting a promising method to increase efficacy or overcome resistance to conventional TKIs [13,14]. However, there is increasing interest in asciminib outside combination with TKIs in clinical practice, for example in our patient who had extensive previous TKI exposure with documented resistance, which could stem from mutations in other genes, and experienced severe cardiovascular toxicity from ponatinib precluding the possibility of re-challenge. Indeed, only 2 patients were treated with TKI combinations in an analysis of asciminib compassionate access programs in relapsed or refractory Ph-positive B-ALL, most of which were in third or fourth line of therapy with a median of three TKIs used before asciminib. In this group, median age was 58 years, half received asciminib monotherapy and a quarter were treated with chemotherapy combinations [15].

Though it has only been studies in a small sample with short median follow up, asciminib monotherapy or chemotherapy combinations appear to achieve high rates of hematologic remission [15]. Combination with BiTEs and ADCs may be better poised to achieve deeper molecular responses and can be a viable alternative in chemotherapy ineligible patients as in our case. However, data are scarce, with only two reported patients who received asciminib in combination with chemotherapy and inotuzumab ozogamicin as a bridge to allogeneic HSCT [X] and an ongoing phase 2 clinical trial of asciminib in combination with blinatumomab (NCT06308588). Herein, we showed the high effectiveness of a combination without a TKI or chemotherapy backbone, building on the role of targeted therapy. Despite its cytotoxic activity, inotuzamab ozogamicin is well-tolerated in the elderly population [16] but single agent use effectiveness in maintaining long-term disease control is limited [17]. Thus, combination with asciminib may represent a new avenue to enhance responses.

Patients with multiply relapsed Ph-positive ALL that have exhausted most TKIs, BiTE, and CAR T-cell therapy have very limited treatment options. This situation is particularly challenging in older adults who are not candidates for cytotoxic chemotherapy or allogeneic HSCT due to medical comorbidity. Herein, we describe a patient in this situation who achieved durable MRD-negative CR following successful treatment with a combination of asciminib and inotuzumab ozogamicin. The patient had extensive previous exposure to multiple TKIs with severe TKI-induced adverse events and an ABL1 KD mutation. Outside of hematologic toxicity, which was manageable with cycle delays, the treatment was well tolerated despite elderly age, demonstrating a novel, safe and effective treatment avenue for highly refractory Ph-positive B-ALL. Our strategy focused on combining asciminib with targeted therapy, in this case targeting CD22, which is a novel therapeutic approach that offers relevant advantages in real-world clinical practice scenarios, though further studies are needed to optimize dosing regimens and synergistic effect, while minimizing toxicity. To our knowledge, this is the first report to date of a patient successfully treated with the asciminib and inotuzumab ozogamicin combination without a conventional TKI or a chemotherapy backbone. The deep MRD-negative CR achieved with this combination can be a bridge to subsequent allogeneic HSCT in eligible patients [4] (Fig. 1).

**Fig. 1:**
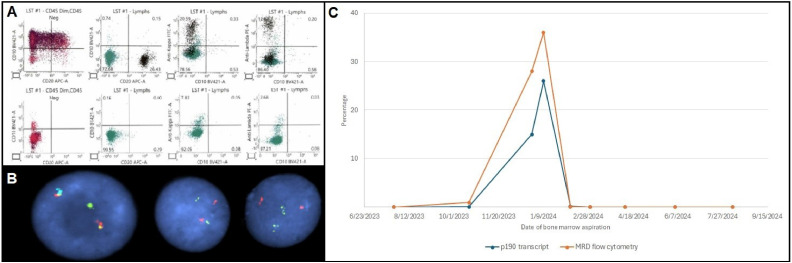
A. Upper: January 2024 flow cytometry from bone marrow aspirate showing CD45dim cells expressing blasts markers (first panel) and mature B-cell markers (rest of panels). Lower: March 2024 flow cytometry from bone marrow aspirate showing absent cells with the abnormal immunophenotype previously detected (first panel) and essentially absent B cells (rest of panels). B. BCR/ABL1 dual color, dual fusion FISH (Cytocell); interphase FISH with 2 fusion signals in bone marrow aspirate sample from December 2014 (to the left) and normal comparison (in sample post treatment, in remission). C. Quantified BCR::ABL1 p190 transcript and measurable residual disease by multiparameter flow cytometry over time before and after treatment with asciminib and inotuzumab ozogamicin in third relapse.

## CRediT authorship contribution statement

**Aneta Strumilowska:** Writing – review & editing, Writing – original draft, Data curation, Conceptualization. **Katherine Devitt:** Visualization, Validation, Data curation. **Joanna Conant:** Visualization, Formal analysis, Data curation, Conceptualization. **Juli-Anne Gardner:** Visualization, Data curation, Conceptualization. **Ashley Volaric:** Visualization, Data curation, Conceptualization. **Neel Hegde:** Visualization, Data curation. **Diego Adrianzen-Herrera:** Writing – review & editing, Writing – original draft, Investigation, Data curation, Conceptualization.

## Declaration of competing interest

None of the authors have any financial benefits or conflicts of interest to disclose.
